# Relationships between tumour response and primary tumour location, and predictors of long-term survival, in patients with *RAS* wild-type metastatic colorectal cancer receiving first-line panitumumab therapy: retrospective analyses of the PRIME and PEAK clinical trials

**DOI:** 10.1038/s41416-018-0165-z

**Published:** 2018-07-17

**Authors:** Marc Peeters, Timothy Price, Julien Taieb, Michael Geissler, Fernando Rivera, Jean-Luc Canon, George Pentheroudakis, Reija Koukakis, Peter Burdon, Salvatore Siena

**Affiliations:** 1 Department of Oncology, Antwerp University Hospital, Wilrijkstraat 10, 2650 Edegem, Belgium; 2 Queen Elizabeth Hospital/University of Adelaide, 28 Woodville Rd, Woodville South, SA, 5011 Australia; 3 Sorbonne Paris Cité, Paris Descartes University, Georges Pompidou European Hospital, 20 Rue Leblanc, 75015 Paris, France; 4 Klinikum Esslingen, Cancer Center Esslingen, Hirschlandstraße 97, 73730 Esslingen am Neckar, Germany; 5 Hospital Universitario Marqués de Valdecilla, Av. Valdecilla, 25, 39008 Santander, Spain; 6 Service d’Oncologie-Hématologie, Grand Hôpital de Charleroi, Avenue du Centenaire 73, 6061 Charleroi, Belgium; 7 Department of Medical Oncology, Ioannina University Hospital, Leof. Stavrou Niarchou, 455 00 Ioannina, Greece; 8 Biostatistics, Amgen Ltd, Sanderson Road, Uxbridge, UB8 1DH UK; 9 European Medical, Amgen (Europe) GmbH, Dammstrasse 23, 6300 Zug, Switzerland; 10 Dipartimento di Oncologia e Emato-Oncologia, Università degli Studi di Milano, Niguarda Cancer Center, Grande Ospedale Metropolitano Niguarda, Piazza dell’Ospedale Maggiore, 3, 20162 Milan, Italy

**Keywords:** Targeted therapies, Targeted therapies

## Abstract

**Background:**

Data from two trials of panitumumab in metastatic colorectal cancer (mCRC) were retrospectively analysed to investigate the effects of primary tumour location on early-tumour shrinkage (ETS) and depth of response (DpR), and identify factors predicting long-term survival.

**Methods:**

Patients with *RAS* wild-type mCRC from PRIME (NCT00364013) and PEAK (NCT00819780) were included. ETS was defined as a ≥30% reduction in the sum-of-the-longest-diameters of measurable target lesions at eight weeks. DpR was the maximum percentage change from baseline to nadir in patients with shrinkage. Univariate and multivariate logistic analyses of short- versus long-term survivor data were performed.

**Results:**

A total of 435/559 (78%) patients had left-sided disease. Of these, a higher proportion of patients treated with panitumumab versus comparator achieved ETS (PRIME: 62% vs. 36%; PEAK: 58% vs. 41%); median DpR was also higher with panitumumab (PRIME: 59% vs. 49%; PEAK: 70% vs. 48%). In pooled analyses of the studies, more patients with right-sided disease achieved ETS if treated with panitumumab than comparator (39% vs. 29%). Panitumumab treatment consistently predicted long-term survival.

**Conclusions:**

First-line panitumumab was associated with improved ETS and DpR vs. comparator in patients with left-sided mCRC. ETS may identify a subgroup of patients with right-sided disease who might respond to panitumumab.

## Introduction

Colorectal cancer (CRC) is one of the leading causes of cancer deaths worldwide.^1^ Almost 50% of patients with CRC develop metastatic disease, with 25% possessing distant metastases at the time of diagnosis.^2^ Systemic treatments for metastatic CRC (mCRC) include conventional fluoropyrimidine-based chemotherapy and biological agents that target vascular angiogenesis or the epidermal growth factor receptor (EGFR).^3,4^ The latter group of targeted agents include the anti-EGFR monoclonal antibodies panitumumab and cetuximab. Patients whose tumours harbour mutations in *RAS* do not respond to EGFR-targeted agents, and may in fact experience inferior outcomes if such agents are combined with oxaliplatin-containing chemotherapy regimens.^4–6^ It is therefore essential that before anti-EGFR therapy is initiated in a patient with mCRC, *RAS* wild-type (WT) status is confirmed.^3,4^ V600E *BRAF* mutations—present in ~10% of CRC tumours—are a negative prognostic marker in patients with mCRC, although current evidence on whether these mutations are predictive of the response to anti-EGFR agents is inconclusive.^3,4,7–9^ Furthermore, unlike *RAS*, extended (non-V600E) *BRAF* mutations may not have the same clinical implications.^10^ Additional biomarkers that may impact on the response to EGFR-targeted therapies, but remain to be confirmed, include *human epidermal growth factor (HER) 2* and *3* and *MET* gene amplification.^4,11–13^ For example, studies have indicated that *HER2* activation substitutes for EGFR dependence in a subset of mCRC patients, and is therefore a potential negative predictor of benefit to anti-EGFR therapy.^12^

Another factor known to affect prognosis and treatment outcomes in mCRC is primary tumour location. Right-sided tumours are less prevalent and associated with a poorer prognosis than left-sided tumours.^14–17^ Right-sided tumours are also more frequently associated with mutations in *BRAF, TGFβR2* and *PI3KCA*.^18,19^ In contrast, amplification of EGFR and HER2, overexpression of EGFR ligands and chromosomal instability are more common in left- than right-sided tumours.^18–20^ According to the CRC Subtyping Consortium, right-sided tumours are more likely to be consensus molecular subtype 1 (CMS1), while left-sided tumours are predominantly CMS2.^21^

In exploratory analyses of two randomised controlled trials, PEAK and PRIME, tumour location was found to influence outcomes following treatment with the anti-EGFR monoclonal antibody, panitumumab.^14^ In the phase II PEAK study, patients with *RAS* WT left-sided disease treated with panitumumab plus modified (m)FOLFOX6 had numerically longer median PFS and median overall survival (OS) than patients treated with bevacizumab plus mFOLFOX6 (PFS: 14.6 vs. 11.5 months; OS: 43.4 vs. 32.0 months). In the larger phase III PRIME study, panitumumab plus FOLFOX4 significantly increased median PFS and median OS vs. FOLFOX4 alone in *RAS* WT patients with left-sided tumours (PFS: 12.9 vs. 9.2 months; OS: 30.3 vs. 23.6 months). For patients with right-sided tumours, however, no statistically significant differences in PFS or OS between panitumumab and the comparator arms were observed in either PRIME or PEAK, although no definitive conclusions could be drawn due to the relatively low number of these patients.^14^ In both trials, response rates were higher in the panitumumab arm vs. the comparator arm for both left- and right-sided *RAS* WT tumours. Analyses of other panitumumab studies showed that patients with *RAS* WT left-sided mCRC also benefit when this agent is provided as a second-line treatment. Meta-analyses of randomised controlled trials in mCRC have shown improved OS and PFS in *RAS* WT patients with left-sided tumours—but not in those with right-sided tumours—who were treated with EGFR-targeted antibodies plus chemotherapy, relative to chemotherapy alone or chemotherapy plus bevacizumab.^22,23^ In the same meta-analyses, however, numerical increases in ORR were observed in patients with right-sided tumours who received anti-EGFR treatment (as well as in those with left-sided tumours).^22,23^ These ORR findings indicate that doublet chemotherapy plus anti-EGFR therapy remains an option for patients with right-sided tumours in whom cytoreduction is the goal.

Here we report additional analyses of the PRIME and PEAK studies. The first set of these analyses were performed to further characterise the effect of primary tumour location and panitumumab treatment on previously untreated patients with *RAS* WT mCRC. Two newer measures of tumour response were considered: early-tumour shrinkage (ETS) and depth of response (DpR). Used increasingly in studies of mCRC, ETS and DpR provide information on tumour shrinkage beyond that provided by the more traditional Response Evaluation Criteria In Solid Tumors (RECIST).^24^ ETS may also offer an early indication of sensitivity to treatment,^25,26^ while DpR reveals the maximum tumour shrinkage achieved.^27^ To date, there are limited data on these response assessments according to primary tumour location. The second set of analyses reported here were performed to build upon previous findings that primary tumour location influences outcomes in the first-line setting. Specifically, we aimed to identify other patient or disease characteristics that may predict good outcomes (long-term survival) in patients with mCRC. To do this, we pooled data across treatment arms from PRIME and PEAK and compared characteristics between patients with, and those without, very extended survival. Preliminary results of these analyses have previously been presented in abstract form.^28,29^

## Methods

### Study designs

Both PRIME and PEAK were randomised controlled trials that recruited patients with previously untreated mCRC. PRIME (ClinicalTrials.gov identifier: NCT00364013) was a phase III study that compared the efficacy and safety of panitumumab 6 mg/kg every two weeks (Q2W) plus FOLFOX4 with FOLFOX4 alone.^30^ PEAK (NCT00819780) was a phase II study of mFOLFOX6 combined with panitumumab 6 mg/kg Q2W or bevacizumab 5 mg/kg Q2W.^31^ The current analyses focused on *RAS* WT mCRC and therefore only included data from patients whose tumours contained no mutations in *KRAS* or *NRAS* exons 2 (codons 12/13), 3 (codons 59/61) and 4 (codons 117/146). Patients were characterised as having *BRAF* mutations if mutations in *BRAF* exon 15 (at codon 600) were found. All procedures in PRIME and PEAK were carried out in accordance with the 1964 Helsinki declaration and its later amendments or comparable ethical standards, and with the ethical standards of the relevant institutional and/or national research committees. Signed informed consent was obtained from all patients. Separate consent was not required for these retrospective analyses.

### Analyses

#### Impact of primary tumour location on outcomes

Information on primary tumour location was obtained from free-text surgery descriptions that were included in case report forms, and from original pathology reports. Primary tumours located in the caecum to transverse colon were coded as right-sided, while tumours located from the splenic flexure to rectum were considered left-sided. Baseline patient demographics and disease characteristics, duration of study drug exposure and post-progression (i.e. post-study) anti-cancer therapy were summarised by study, study treatment and primary tumour location. The effects of primary tumour location on ETS and DpR were retrospectively assessed in each study and in pooled analyses of the two studies. ETS was defined as a reduction of ≥30% in the sum-of-the-longest-diameters of measurable target lesions at eight weeks after initiation of study treatment. A cut-off of ≥30% was selected as this is in line with RECIST criteria and has been associated with a similar pattern of benefit as a ≥20% cut-off.^24^ DpR was calculated as the maximum percentage change from baseline to nadir in patients with tumour shrinkage. In patients with tumour growth or no change in tumour size, DpR was defined as the percentage change from baseline to progression if the patient subsequently progressed, or as ‘missing’ if the patient did not progress. Thus, DpR was positive if there was shrinkage, negative if there was growth and zero if there was no change. Other outcomes assessed in the single study and/or pooled analyses were PFS, OS, objective response rate and resection rate. A Cox proportional hazard model was used to calculate hazard ratios (HRs) and 95% confidence intervals (CI) for PFS, OS and ETS. Significance was determined using the Wilcoxon rank-sum test. The Kaplan–Meier method was used to analyse PFS and OS by treatment, primary tumour location and ETS status.

#### Characteristics and response outcomes of long-term survivors

Long-term survivors were identified using two different definitions: patients with OS ≥45 months or the 25% of patients with the longest OS. Given that OS in mCRC is reported to be ≥30 months, 45 months was chosen as a reasonable cut-off for long-term survival.^32,33^ For validation, the 25% of patients with the longest OS were also analysed. Baseline demographic and disease characteristics, study exposure and outcomes of first-line therapy were summarised for each study. Based on the work of Köhne and colleagues,^34^ patients were retrospectively assigned a baseline prognostic score (‘Köhne score’) that classified subjects into high-, medium- and low-risk groups based on four baseline clinical parameters: Eastern Cooperative Oncology Group (ECOG) performance status, white blood cell count, alkaline phosphatase level and number of metastatic sites. ETS and DpR were also assessed, defined as above. Univariate logistic analyses were performed to descriptively assess the potential relationship of different covariates with long-term survival. The covariates included were sites of metastases, sex, treatment, ECOG performance status, *BRAF* status, primary tumour location, prior adjuvant chemotherapy, country, age, Köhne score and disease stage. A multivariate logistic analysis was then conducted using a stepwise model-building procedure with a 10% significance level for a covariate to enter or remain in the model. Patients who were censored before the cut-off date for the long-term survivor analyses were excluded from these analyses as it was not known if such patients were long- or short-term survivors (by either definition of long-term survival).

## Results

### Impact of primary tumour location on outcomes

#### Patients, study drug exposure and post-progression anti-cancer therapy

As previously reported,^14^ there were 675 patients in the *RAS* WT populations of PRIME and PEAK. Among the 559 patients for whom primary tumour location could be determined, 435 (78%) had left-sided tumours and a lower proportion, 124 (22%), had right-sided tumours, reflective of the typical distribution in *RAS* WT populations. In both studies, patient demographics and disease characteristics at baseline were generally similar between patients with left- and right-sided disease, although those with right-sided disease were more likely to have *BRAF* mutations (Table 1). In PRIME but not PEAK, right-sided disease was more frequent in females than in males.

**Table 1:** Baseline patient demographics and disease characteristics, study drug exposure and post-progression anti-cancer therapy by treatment and primary tumour location in PRIME and PEAK

	PRIME				PEAK			
	Panitumumab + FOLFOX4		FOLFOX4		Panitumumab + mFOLFOX6		Bevacizumab + mFOLFOX6	
	Left (*n* = 169)	Right (*n* = 39)	Left (*n* = 159)	Right (*n* = 49)	Left (*n* = 53)	Right (*n* = 22)	Left (*n* = 54)	Right (*n* = 14)
*Baseline demographics/disease characteristics*								
Median age (range), years	61 (27–81)	62 (42–80)	62 (27–82)	61 (24–78)	60 (23–77)	64 (43–82)	60 (39–82)	66 (50–78)
Sex, n (%)								
Male	120 (71)	21 (54)	103 (65)	25 (51)	34 (64)	15 (68)	38 (70)	10 (71)
Female	49 (29)	18 (46)	56 (35)	24 (49)	19 (36)	7 (32)	16 (30)	4 (29)
BRAF status^a,b^, *n* (%)								
Mutant	7 (4)	13 (33)	8 (5)	16 (33)	1 (2)	9 (41)	1 (2)	1 (7)
Wild-type	156 (92)	26 (67)	148 (93)	32 (65)	52 (98)	13 (59)	53 (98)	13 (93)
Site of metastases, *n* (%)								
Liver + other	119 (70)	21 (54)	108 (68)	35 (71)	21 (40)	13 (59)	21 (39)	9 (64)
Liver only	33 (20)	6 (15)	31 (19)	5 (10)	18 (34)	4 (18)	15 (28)	4 (29)
Other only	17 (10)	12 (31)	20 (13)	9 (18)	14 (26)	5 (23)	18 (33)	1 (7)
ECOG performance status^a^, *n* (%)								
0	106 (63)	22 (56)	88 (55)	27 (55)	37 (70)	10 (45)	35 (65)	9 (64)
1	56 (33)	15 (38)	61 (38)	19 (39)	16 (30)	12 (55)	19 (35)	5 (36)
2	7 (4)	2 (5)	9 (6)	3 (6)	0	0	0	0
Stage IV disease at diagnosis, *n* (%)	101 (60)	21 (54)	90 (57)	23 (47)	28 (53)	14 (64)	27 (50)	10 (71)
Prior adjuvant chemotherapy, *n* (%)	29 (17)	10 (26)	26 (16)	10 (20)	8 (15)	4 (18)	13 (24)	4 (29)
*Study drug exposure and post-progression anti-cancer therapy*								
First-line study drug exposure, *n* (%)								
<3 months	20 (12)	9 (23)	23 (14)	12 (24)	5 (9)	3 (14)	11 (20)	5 (36)
≥3 to <6 months	45 (27)	13 (33)	48 (30)	16 (33)	13 (25)	7 (32)	13 (24)	1 (7)
≥6 to <9 months	33 (20)	5 (13)	44 (28)	11 (22)	10 (19)	6 (27)	11 (20)	3 (21)
≥9 months	71 (42)	12 (31)	44 (28)	10 (20)	25 (47)	6 (27)	19 (35)	5 (36)
Any post-PD anti-cancer therapy^c^, *n* (%)	126 (75)	21 (54)	120 (75)	32 (65)	39 (74)	14 (64)	44 (81)	9 (64)
Any post-PD anti-EGFR mAb	31 (18)	3 (8)	42 (26)	13 (27)	18 (34)	5 (23)	27 (50)	7 (50)
Small molecule EGFRi	1 (1)	0	4 (3)	0	0	1 (5)	0	0
Any post-PD anti-VEGF therapy	35 (21)	7 (18)	32 (20)	10 (20)	28 (53)	10 (45)	23 (43)	4 (29)
Fluoropyrimidine	97 (57)	17 (44)	89 (56)	22 (45)	37 (70)	13 (59)	34 (63)	4 (29)
Irinotecan	94 (56)	17 (44)	97 (61)	29 (59)	34 (64)	10 (45)	36 (67)	7 (50)
Oxaliplatin	35 (21)	6 (15)	25 (16)	6 (12)	13 (25)	1 (5)	13 (24)	3 (21)
Other/unknown	74 (44)	12 (31)	62 (39)	17 (35)	31 (58)	10 (45)	41 (76)	4 (29)

*ECOG* Eastern Cooperative Oncology Group, *EGFR(i)* epidermal growth factor receptor (inhibitor), *mAb* monoclonal antibody, *mFOLFOX6* modified FOLFOX6, *PD* progressive disease, *VEGF* vascular endothelial growth factor.

^a^Unknown for some patients.

^b^Patients were characterised as having *BRAF* mutations if mutations in *BRAF* exon 15 (at codon 600) were found.

^c^Patients could have ≥1 post-PD therapy

More patients with left-sided tumours than right-sided tumours received study drug for at least nine months. This was the case in both PRIME and PEAK (Table 1) and when data were pooled across the two studies (159/435 (37%) vs. 33/124 (27%), respectively). Post-progression anti-cancer therapy was received by a greater proportion of patients with left-sided than right-sided disease (PRIME: 246/328 (75%) vs. 53/88 (60%); PEAK: 83/107 (78%) vs. 23/36 (64%)). In general, a similar trend was observed for each of the different treatments that were provided after progression (Table 1). Among patients treated with panitumumab, those with left-sided disease were more likely to receive post-progression anti-EGFR therapy than those with right-sided disease in both PRIME and PEAK. In contrast, among patients treated with comparator (FOLFOX4 alone in PRIME; mFOLFOX6 plus bevacizumab in PEAK), there were no differences in the proportion of patients with left- vs. right-sided disease who received post-progression anti-EGFR therapy (Table 1).

#### Outcomes—single study analyses

Table 2 shows, for PRIME and PEAK, the effects of panitumumab and comparator treatment on patient outcomes—including ETS and DpR—by primary tumour location. In patients with left-sided disease, ETS was achieved in a higher proportion of those who received panitumumab than those who received comparator (PRIME 62% vs. 36%; PEAK: 58% vs. 41%). In patients with right-sided disease, a similar proportion achieved ETS with panitumumab and comparator in PRIME (31% in both treatment arms). In PEAK, however, a greater proportion of these patients experienced ETS with panitumumab than with comparator (55% vs. 21%). For patients with left-sided tumours, median DpR was higher with panitumumab than with comparator (PRIME: 59% vs. 49%, PEAK: 70% vs. 48%). The effects of treatment on DpR were less clear in patients with right-sided disease (PRIME: 37% vs. 50%; PEAK: 50% vs. 45%). In both studies, more patients with left- than right-sided disease underwent any resection and complete (R0) resection (Table 2).

**Table 2:** Outcomes by treatment and primary tumour location in PRIME and PEAK

Characteristic	PRIME				PEAK			
	Panitumumab + FOLFOX4		FOLFOX4		Panitumumab + mFOLFOX6		Bevacizumab + mFOLFOX6	
	Left (*n* = 169)	Right (*n* = 39)	Left (*n* = 159)	Right (*n* = 49)	Left (*n* = 53)	Right (*n* = 22)	Left (*n* = 54)	Right (*n* = 14)
Median PFS (95% CI), months	12.9 (10.0–14.6)	7.5 (5.5–10.4)	9.2 (7.6–10.7)	7.0 (5.4–8.0)	14.6 (11.6–17.7)	8.7 (5.7–10.9)	11.5 (9.3–13.0)	12.6 (1.8–16.6)
Median OS (95% CI), months	30.3 (25.8–36.1)	11.1 (8.1–25.2)	23.6 (18.2–26.9)	15.4 (9.1–21.7)	43.3 (31.6–63.0)	17.5 (9.1–30.7)	32.0 (26.0–47.4)	21.0 (6.0–29.0)
Objective response rate^a^, *n*/*N* (%)	114/168 (68)	16/38 (42)	82/156 (53)	16/46 (35)	34/53 (64)	14/22 (64)	31/54 (57)	7/14 (50)
ETS^b^ ≥30%, *n* (%)	104 (62)	12 (31)	57 (36)	15 (31)	31 (58)	12 (55)	22 (41)	3 (21)
Median PFS (95% CI), months	14.8 (12.5–18.5)	14.9 (7.4–27.2)	11.1 (9.3–13.9)	7.3 (5.6–11.1)	16.2 (13.0–20.3)	10.8 (5.5–15.8)	12.9 (9.3–18.6)	18.4 (16.6–21.4)
Median OS (95% CI), months	35.0 (29.8–41.9)	27.2 (8.0–57.4)	31.7 (23.8–38.1)	23.6 (7.2–34.5)	55.4 (41.3–63.0)	24.6 (10.3–48.4)	48.5 (28.9–NE)	26.2 (21.0–31.3)
ETS^b^ <30%, *n* (%)	49 (29)	22 (56)	87 (55)	27 (55)	20 (38)	7 (32)	28 (52)	9 (64)
Median PFS (95% CI), months	9.4 (5.8–13.8)	6.5 (4.0–9.9)	6.9 (5.5–7.8)	6.9 (3.6–11.9)	11.6 (7.5–16.4)	5.8 (3.6–9.8)	12.4 (7.4–13.0)	12.6 (1.8–13.8)
Median OS (95% CI), months	19.9 (13.5–27.5)	10.6 (6.1–22.5)	17.2 (14.2–20.7)	13.1 (6.1–18.8)	34.2 (17.3–48.0)	15.3 (5.8–46.1)	27.7 (21.0–32.0)	23.3 (6.0–29.0)
Median DpR (IQR), %	59^*^ (37–73)	37^*^ (12–52)	49 (27–67)	50 (21–65)	70^*^ (47–100)	50^*^ (23–70)	48 (29–80)	45 (16–56)
Any resection, *n* (%)	25 (15)	4 (10)	21 (13)	6 (12)	9 (17)	2 (9)	10 (19)	1 (7)
R0 resection, *n* (%)	19 (11)	2 (5)	16 (10)	1 (2)	7 (13)	1 (5)	6 (11)	1 (7)
Any resection—LLD population, *n*/*N* (%)	10/33 (30)	2/6 (33)	8/31 (26)	2/5 (40)	5/18 (28)	1/4 (25)	6/15 (40)	1/4 (25)
R0 resection—LLD population, *n*/*N* (%)	9/33 (27)	2/6 (33)	6/31 (19)	0/5 (0)	4/18 (22)	1/4 (25)	5/15 (33)	1/4 (25)

*CI* confidence interval, *DpR* depth of response, *ETS* early-tumour shrinkage, *IQR* interquartile range, *LLD* liver-limited disease, *mFOLFOX6* modified FOLFOX6, *NE* not evaluable, *OS* overall survival, *PFS* progression-free survival, *R0* complete resection.

^*^p-value for difference between tumour side <0.05.

^a^Denominator is the number of patients evaluable for response.

^b^ETS status was unknown for some patients

#### Outcomes–pooled analyses of PRIME and PEAK

**Fig. 1:**
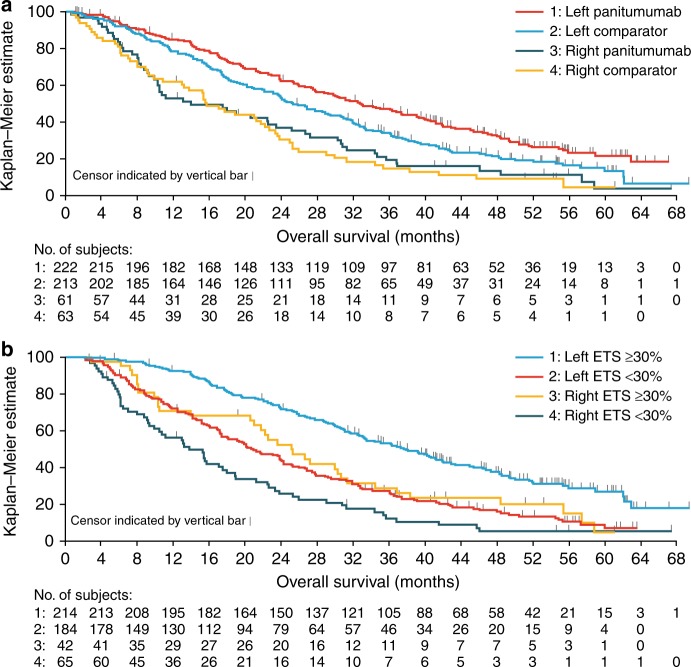
Kaplan–Meier plots showing impact of **a** primary tumour location and treatment on OS, and **b** primary tumour location and ETS on OS in pooled analyses from PRIME and PEAK. ETS early-tumour shrinkage, OS overall survival

In pooled analyses of PRIME and PEAK, panitumumab improved PFS in patients with left-sided disease compared with comparator treatment (HR: 0.70, 95% CI: 0.58–0.86). OS was also improved with panitumumab vs. comparator in patients with left-sided disease (HR: 0.71, 95% CI: 0.57–0.89; Fig. 1a). In patients with right-sided disease, the effects of panitumumab on PFS and OS relative to comparator were less clear (HR for PFS: 0.84, 95% CI: 0.58–1.23; HR for OS: 0.90, 95% CI: 0.61–1.32).

In a pooled analysis of both studies and all treatment arms, more patients with left- vs. right-sided disease experienced ETS (214/435 (49%) vs. 42/124 (34%)). In total, 39% (24/61) of patients with right-sided tumours who received panitumumab experienced ETS. In these patients, median PFS was 10.9 months (95% CI: 9.0–15.8) and median OS was 26.6 months (95% CI: 10.4–36.9). Among patients with right-sided disease who received comparator treatment, 29% (18/63) had ETS; these patients had a median PFS of 9.4 months (95% CI: 6.2–16.6) and median OS of 23.6 months (95% CI: 9.8–34.5). Pooled analysis across studies and treatments showed that ETS was associated with improved PFS compared with ETS <30%, irrespective of primary tumour location (HR in left-sided disease: 0.57, 95% CI: 0.46–0.70; HR in right-sided disease: 0.60, 95% CI: 0.39–0.91). Similar results were seen for the impact of ETS on OS (HR in left-sided disease: 0.48, 95% CI: 0.38–0.60; HR in right-sided disease: 0.58, 95% CI: 0.38–0.89; Fig. 1b). Across both studies and all treatments, more patients with left- than right-sided disease underwent any resection (65/435 (15%) vs. 12/124 (10%)) or complete (R0) resection (48/435 (11%) vs. 5/124 (4%)).

### Characteristics and response outcomes of long-term survivors

#### Patients

A total of 612 patients were included in the long-term survivor analysis. Baseline demographics and disease characteristics for long- and short-term survivors (defined using the 45-month or 25% cut-offs) are shown for each study in Supplementary Table 1. In both studies, long-term survivors defined using the 45-month cut-off were more likely than short-term survivors to have been treated with panitumumab, have an ECOG performance status of 0, have *BRAF* WT mCRC, have left-sided primary disease and have liver-only metastases. Similar trends were observed when long-term survival was defined using the 25% cut-off (Supplementary Table 1).

#### Outcomes during first-line therapy in long-term survivors

Responses of long- and short-term survivors (defined according to both cut-offs) are summarised in Table 3. Using the 25% cut-off, median OS irrespective of treatment arm was 39.5 months in PRIME (120/475 patients) and 45.9 months in PEAK (34/137 patients). The 25% of patients with the highest OS were more likely, compared with short-term survivors, to have received study treatment for at least 9 months, achieved an objective complete or partial response, undergone any or complete resection, and experienced ETS (Table 3). These results were driven by those patients with left-sided tumours (Supplementary Table 2). Median DpR was also higher in these long-term survivors. Across PRIME and PEAK, 124 patients (21%) survived for ≥45 months (Table 3). In pooled analysis of the studies using the 45-month cut-off, median DpR was 79% (interquartile range: 61–100) in long-term survivors and 44% (22–61) in short-term survivors, with 68% (84/124) and 37% (175/478) of patients, respectively, experiencing ETS.

**Table 3:** Response to first-line therapy in long- and short-term survivors in PRIME and PEAK

Characteristic	25% survival cut-off^a^				45-month cut-off^b^			
	PRIME		PEAK		PRIME		PEAK	
	Long-term survivor (*n* = 120)	Short-term survivor (*n* = 355)	Long-term survivor (*n* = 34)	Short-term survivor (*n* = 103)	Long-term survivor (*n* = 89)	Short-term survivor (*n* = 375)	Long-term survivor (*n* = 35)	Short-term survivor (*n* = 103)
Exposure duration, *n* (%)								
<3 months	5 (4)	72 (20)	4 (12)	15 (15)	5 (6)	72 (19)	4 (11)	15 (15)
≥3 to <6 months	21 (18)	110 (31)	6 (18)	28 (27)	14 (16)	113 (30)	6 (17)	28 (27)
≥6 to <9 months	27 (23)	83 (23)	7 (21)	23 (22)	21 (24)	86 (23)	8 (23)	23 (22)
≥9 months	67 (56)	90 (25)	17 (50)	37 (36)	49 (55)	104 (28)	17 (49)	37 (36)
Resection, *n* (%)								
Any	36 (30)	21 (6)	9 (26)	3 (3)	29 (33)	22 (6)	10 (29)	3 (3)
Complete	29 (24)	9 (3)	6 (18)	3 (3)	23 (26)	10 (3)	6 (17)	3 (3)
Best overall response, *n* (%)								
Complete response	0	2 (1)	5 (15)	0	0	2 (1)	5 (14)	0
Partial response	89 (74)	160 (45)	24 (71)	59 (57)	67 (75)	174 (46)	24 (69)	59 (57)
Stable disease	25 (21)	136 (38)	5 (15)	32 (31)	16 (18)	142 (38)	6 (17)	32 (31)
Progressive disease	4 (3)	44 (12)	0	5 (5)	4 (4)	44 (12)	0	5 (5)
Not known/unavailable	2 (2)	13 (4)	0	7 (7)	2 (2)	13 (3)	0	7 (7)
ETS, *n* (%)	77 (64)	123 (35)	26 (76)	42 (41)	58 (65)	133 (35)	26 (74)	42 (41)
Median DpR (IQR), %	75 (60–89)	43 (18–59)	86 (61–100)	45 (26–60)	77 (61–96)	44 (19–62)	87 (61–100)	45 (26–60)
Received post-progression EGFRi therapy, *n* (%)	44 (37)	62 (17)	16 (47)	40 (39)	30 (34)	71 (19)	17 (49)	40 (39)

*DpR* depth of response, *EGFRi* epidermal growth factor receptor inhibitor, *ETS* early-tumour shrinkage, *IQR* interquartile range, *OS* overall survival.

^a^Long-term survival defined as the 25% of classifiable patients with the longest OS (this included some patients that survived less than 45 months); all other patients were defined as short-term survivors.

^b^Long-term survival defined as OS ≥45 months; short-term survival defined as OS <45 months

The covariates included in the descriptive univariate analysis, and the results of this analysis when the 45-month cut-off was used, are shown in Supplementary Table 3. In multivariate analyses, panitumumab treatment (vs. comparator) was the only factor that significantly predicted long-term survival in both PRIME and PEAK according to both the 45-month cut-off (Table 4) and the 25% cut-off (data not shown). In PRIME, medium-risk Köhne score, liver-only metastases, ECOG performance status 0 (25% cut-off only), *BRAF* WT status, study location in Western Europe, Canada or Australia (45-month cut-off only), age < 65 years (45-month cut-off only) and stage I disease (45-month cut-off only) were also significantly associated with long-term survival. In PEAK, female sex, left-sided primary tumour location, ECOG performance status 0 and prior adjuvant chemotherapy were associated with long-term survival (in addition to panitumumab treatment). In pooled multivariate analyses of PRIME and PEAK using the 45-month cut-off, panitumumab treatment again predicted long-term survival, along with low- and medium-risk Köhne score, *BRAF* WT status and left-sided primary tumour location (Table 4).

**Table 4:** Independent predictors of long-term overall survival in multivariate analysis in PRIME and PEAK (45-month cut-off for long-term survival)

	Odds ratio (95% CI)^a^
*PRIME*	
Köhne score	
Low risk vs. high risk	3.00 (0.33–27.49)
Medium risk vs. high risk	6.26 (2.15–18.29)
Treatment: FOLFOX4 vs. panitumumab + FOLFOX4	0.50 (0.30–0.83)
Sites of metastases	
Liver + other vs. liver only	0.28 (0.04–2.26)
Other only vs. liver only	0.11 (0.01–0.85)
BRAF status	
Mutant vs. wild-type	0.08 (0.01–0.63)
Missing vs. wild-type	1.58 (0.37–6.80)
Region: Rest of the world vs. Western Europe, Canada or Australia	0.56 (0.32–0.96)
Age (≥65 years): No vs. yes	1.69 (0.98–2.91)
Stage	
I–III vs. IV	2.37 (1.29–4.36)
Missing vs. IV	1.04 (0.20–5.46)
*PEAK*	
Treatment: mFOLFOX6 + bevacizumab vs. mFOLFOX6 + panitumumab	0.36 (0.15–0.89)
Sex: Female vs. male	2.93 (1.17–7.34)
Primary tumour location	
Right vs. left	0.16 (0.04–0.63)
Unknown vs. left	0.25 (0.05–1.26)
Prior adjuvant chemotherapy: No vs. yes	0.37 (0.13–1.08)
ECOG performance status: 1 vs. 0	0.29 (0.11–0.81)
*PRIME and PEAK*	
Köhne score	
Low risk vs. high risk	7.85 (2.93–21.03)
Medium risk vs. high risk	5.61 (2.18–14.42)
Treatment: Comparator vs. panitumumab	0.51 (0.33–0.77)
BRAF status	
Mutant vs. wild-type	0.07 (0.01–0.54)
Missing vs. wild-type	0.94 (0.24–3.73)
Side of tumour	
Right vs. left	0.49 (0.25–0.94)
Unknown vs. left	0.48 (0.25–0.94)

*CI* confidence interval, *ECOG* Eastern Cooperative Oncology Group, *mFOLFOX6* modified FOLFOX6.

^a^A stepwise model-building procedure was used with a 10% significance level for a covariate to enter or remain in the model. The table shows all covariates that were significant at the 10% significance level in the final model. An odds ratio >1 indicates that long-term survival is more likely for the first parameter listed, while an odds ratio <1 indicates that long-term survival is more likely for the second parameter listed

## Discussion

We performed two new sets of retrospective analyses of PRIME and PEAK, two randomised controlled trials that recruited patients with previously untreated *RAS* WT mCRC. The main aim of the first set of analyses was to characterise the effects of primary tumour location and panitumumab treatment on patient outcomes in the first-line setting, beyond those already reported.^14^ For patients with left-sided disease, our analyses suggest that in addition to its known beneficial effects on OS, PFS and other ‘traditional’ endpoints in this population, panitumumab may also improve outcomes relative to comparator treatment as measured by two newer endpoints, ETS and DpR. The data were less conclusive for patients with right-sided disease but suggest that ETS may identify a subgroup of these patients who might respond to panitumumab. The second set of analyses, which aimed to identify other patient or disease characteristics that may predict a favourable prognosis in patients with mCRC, found that panitumumab treatment was the only factor that significantly predicted long-term survival in both trials.

Regarding the first set of analyses, individual assessments of PRIME and PEAK revealed that a higher proportion of patients with left-sided disease achieved ETS with panitumumab than with comparator. Median DpR was also higher with panitumumab vs. comparator in these patients in both studies. ETS, as well as DpR, provide information on tumour shrinkage over and above that provided by RECIST, specifically on the timing, depth and duration of response. As a result, they are increasingly being utilised as endpoints in mCRC studies. Achieving early and sustained tumour shrinkage is an important treatment goal in patients with mCRC as it may increase the chance of surgical resection, reduce chemotherapy-associated liver toxicity prior to resection and provide relief of tumour-related symptoms.^26,35^ That our pooled analysis of PRIME and PEAK showed superior survival outcomes with panitumumab vs. comparator in patients with left-sided disease was consistent with other reports.^15–17^ Overall, our findings in patients with previously untreated left-sided *RAS* WT tumours further confirm that anti-EGFR therapy is an appropriate choice for this population. However, the benefits of panitumumab treatment in the PEAK and PRIME studies were less evident in patients with right-sided disease, with DpR, OS and PFS findings, for example, being much less clear in this population. These results are consistent with past reports of an unclear benefit of anti-EGFR therapy in patients with *RAS* WT right-sided mCRC.^14,16,22,23^ Of note, however, objective response rates for right-sided disease have previously been shown to be higher for panitumumab vs. bevacizumab.^14,22^ Moreover, addition of panitumumab to FOLFOXIRI in the randomised phase II VOLFI trial resulted in high response rates in left- and right-sided *RAS* WT mCRC.^36^ It seems pertinent to ask then if there is a subgroup of patients with right-sided disease who should be considered for anti-EGFR therapy and, if so, how they can be identified. With these questions in mind, it is of interest that across the two studies reported here, patients with right-sided disease were more likely to achieve ETS if they were treated with panitumumab than with comparator. Median PFS and OS in patients with right-sided disease who achieved ETS were also numerically longer with panitumumab than with comparator. While intrinsic tumour sensitivity to therapy remains the main determinant of clinical outcome, ETS might therefore identify a subgroup of patients with right-sided disease who may achieve improved outcomes with panitumumab and who may therefore benefit from further treatment. Additional research is needed to help physicians identify such patients in the clinic, including those with bulky tumours and those who can be converted from non-resectable to resectable disease. The optimal treatment approach for these and other mCRC patients also needs to be better defined; for example, with respect to which treatment sequences may be best for different patients. For *RAS* WT patients, accumulating evidence supports the use of first-line anti-EGFR treatment, followed by anti-VEGF therapy.^37,38^ In this context, ETS may be useful in guiding both the decision to switch therapy and the timing of the switch.

Evidence suggests that converting non-resectable mCRC to resectable disease leads to better outcomes.^39^ In the current analyses, patients with left-sided disease were more likely to undergo tumour resection than those with right-sided disease. We also showed that in the panitumumab arms of both PRIME and PEAK, the incidence of post-progression anti-EGFR therapy was higher in patients with left- than right-sided disease. This may be because the former group of patients are more likely to have shown a good response to first-line panitumumab. In patients in the comparator arms, the incidence of post-progression anti-EGFR therapy was similar regardless of primary tumour location. The dynamic susceptibility of mCRC to therapeutic EGFR blockade means that it may be possible to extend survival by rechallenge with EGFR inhibitors. Anti-EGFR treatment may select for treatment-resistant subclones that are present before treatment, leading to secondary resistance of the tumour.^40^ Removing anti-EGFR selection pressure, via a break in anti-EGFR treatment, may allow sensitive clones to become re-established, restoring tumour sensitivity.^41–43^ Rechallenge after a break from EGFR inhibitor treatment may thus be a viable strategy for later lines of treatment in patients with a good initial response.^44,45^ As an early indication of patients who are responding to treatment, ETS might therefore help identify patients suitable for rechallenge. Prospective controlled trials testing rechallenge, and attempting to identify biopsy biomarkers that can predict its efficacy, as well as the mechanisms underlying fluctuating tumour sensitivity to treatment, are currently ongoing, including A-REPEAT (NCT03311750), CHRONOS (NCT03227926) and RASINTRO (NCT03259009).

The second set of analyses reported here aimed to identify clinicopathologic characteristics beyond primary tumour location that may predict a favourable prognosis in patients with *RAS* WT mCRC. These analyses used ‘long-term survival’ as an indicator of good prognosis, defined as either patients with OS of at least 45 months (equivalent to 19% of patients in PRIME and 25% of patients in PEAK) or the 25% of patients with the longest OS. Applying either of these definitions of long-term survival in multivariate analyses, panitumumab treatment was the only factor that significantly predicted long-term survival in both the PRIME and the PEAK trials (and in a pooled analysis of both). Other characteristics that significantly predicted long-term survival in either PRIME or PEAK or both trials combined included baseline disease characteristics associated with favourable prognosis (e.g. low- or medium-risk Köhne score, liver-only metastases, ECOG performance status of 0, *BRAF* WT status), and left-sided disease. Some characteristics could not be modelled in the multivariate analyses due to imbalances across the subgroups, as revealed by univariate analyses. Compared with short-term survivors, long-term survivors more frequently experienced an objective response or ETS, were more likely to have had a resection and had a greater DpR. To our knowledge this is the first reported analysis to compare characteristics of long- and short-term survivors among a cohort of mCRC patients treated with anti-EGFR therapy. Of note, however, ETS has been shown to be associated with longer PFS and OS in patients with *RAS* WT mCRC treated with cetuximab.^25^ Further to this, a retrospective analysis of three trials in *RAS* WT mCRC patients found that ETS and DpR were associated with improved survival.^24^ However, these studies were not designed to compare long- and short-term survivors.

The analyses reported here have provided further insights into the impact of primary tumour location and panitumumab treatment on outcomes in PRIME and PEAK, and on factors beyond left-sided disease that may predict a good prognosis in patients with *RAS* WT mCRC. A limitation of our analyses is that they were post-hoc and unplanned. With respect to ETS and DpR specifically, these endpoints were not in use when the trials were designed ~10 years ago, and thus they were not included as pre-specified endpoints in the trial protocols. ETS and DpR are now, however, used increasingly in mCRC as early indicators of treatment efficacy.^25–27^ A further limitation is that differences in OS were observed between the full study populations of PRIME and PEAK, which may reflect differences in patient characteristics between the two studies (and/or greater data variability in smaller studies such as PEAK or differences in treatment sequencing or use of anti-angiogenic (bevacizumab) therapy). In this respect and due to the differences in trial methodology and the number of patients recruited to each trial, pooled data should be cautiously interpreted. In addition, some patients in the full study populations were omitted from these analyses since data on, for example, tumour mutation status or primary tumour location were not available, or in the case of the long-term survivor analyses, because survival data were censored before the cut-off date. Finally, some of the subgroups analysed, especially those deriving from the PEAK trial and those containing patients with right-sided disease, contained very small numbers of patients that may have impacted on validity of these analyses.

In conclusion, the additional analyses of PRIME and PEAK reported here have shown that first-line panitumumab plus chemotherapy resulted in a higher rate of ETS and a higher DpR compared with first-line chemotherapy (with or without bevacizumab) in *RAS* WT patients with left-sided primary tumours. Pooled analyses also confirmed that panitumumab improved survival outcomes, including PFS and OS, vs. comparator in these patients. In patients with right-sided disease, data were difficult to interpret due to the low patient numbers in this subgroup, yet may indicate that ETS could be used to identify a subgroup of patients with right-sided disease who may achieve improved outcomes with panitumumab therapy. Panitumumab treatment significantly predicted long-term survival in both PRIME and PEAK.

## Electronic supplementary material

Supplementary tables
