# Supplementary material for: Relationships between tumour response and primary tumour location, and predictors of long-term survival, in patients with RAS wild-type metastatic colorectal cancer receiving first-line panitumumab therapy: retrospective analyses of the PRIME and PEAK clinical trials
Source: Br J Cancer. 2018 Jul 17;119(3):303–12. doi: 10.1038/s41416-018-0165-z (PMC6068172; doi:10.1038/s41416-018-0165-z)
Supplement: Supplementary file 1 — Supplementary tables [file 41416_2018_165_MOESM1_ESM.docx]

**Supplementary tables**

| **Supplementary Table 1. Baseline patient demographic and disease characteristics in long-term survivors, defined using 25% survival cut-off or 45-month cut-off, in PRIME and PEAK** | | | | | | | | |
| --- | --- | --- | --- | --- | --- | --- | --- | --- |
| **Characteristic** | **25% survival cut-off^a^** | | | | **45-month cut-off^b^** | | | |
|  | **PRIME** | | **PEAK** | | **PRIME** | | **PEAK** | |
|  | **Long-term survivors (n=120)** | **Short-term survivors (n=355)** | **Long-term survivors (n=34)** | **Short-term survivors (n=103)** | **Long-term survivors (n=89)** | **Short-term survivors (n=375)** | **Long-term survivors (n=35)** | **Short-term survivors (n=103)** |
| Treatment arm |  |  |  |  |  |  |  |  |
| Panitumumab^c^ | 73 (61) | 163 (46) | 21 (62) | 48 (47) | 56 (63) | 173 (46) | 22 (63) | 48 (47) |
| Comparator^d^ | 47 (39) | 192 (54) | 13 (38) | 55 (53) | 33 (37) | 202 (54) | 13 (37) | 55 (53) |
| Median age (range), years | 61 (28–79) | 61 (24–81) | 59 (42–76) | 62 (41–82) | 61 (28–79) | 61 (24–81) | 59 (42–76) | 62 (41–82) |
| Sex, n (%) |  |  |  |  |  |  |  |  |
| Male | 81 (68) | 226 (64) | 18 (53) | 74 (72) | 60 (67) | 240 (64) | 18 (51) | 74 (72) |
| Female | 39 (33) | 129 (36) | 16 (47) | 29 (28) | 29 (33) | 135 (36) | 17 (49) | 29 (28) |
| BRAF status |  |  |  |  |  |  |  |  |
| Mutant | 3 (3) | 49 (14) | 0 | 12 (12) | 1 (1) | 51 (14) | 0 | 12 (12) |
| Wild-type | 113 (94) | 297 (84) | 34 (100) | 91 (88) | 85 (96) | 314 (84) | 35 (100) | 91 (88) |
| Unknown | 4 (3) | 9 (3) | 0 | 0 | 3 (3) | 10 (3) | 0 | 0 |
| Primary tumour location |  |  |  |  |  |  |  |  |
| Left-sided | 92 (77) | 218 (61) | 29 (85) | 56 (54) | 69 (78) | 234 (62) | 30 (86) | 56 (54) |
| Right-sided | 12 (10) | 73 (21) | 3 (9) | 29 (28) | 10 (11) | 74 (20) | 3 (9) | 29 (28) |
| Unknown | 16 (13) | 64 (18) | 2 (6) | 18 (17) | 10 (11) | 67 (18) | 2 (6) | 18 (17) |
| Site of metastases |  |  |  |  |  |  |  |  |
| Liver + other | 72 (60) | 251 (71) | 15 (44) | 51 (50) | 56 (63) | 262 (70) | 15 (43) | 51 (50) |
| Liver only | 37 (31) | 44 (12) | 10 (29) | 22 (21) | 25 (28) | 50 (13) | 10 (29) | 22 (21) |
| Other only | 11 (9) | 60 (17) | 9 (26) | 30 (29) | 8 (9) | 63 (17) | 10 (29) | 30 (29) |
| ECOG performance status |  |  |  |  |  |  |  |  |
| 0 | 89 (74) | 178 (50) | 26 (76) | 58 (56) | 68 (76) | 191 (51) | 27 (77) | 58 (56) |
| 1 | 30 (25) | 148 (42) | 8 (24) | 45 (44) | 21 (24) | 154 (41) | 8 (23) | 45 (44) |
| 2 | 1 (1) | 28 (8) | 0 | 0 | 0 | 29 (8) | 0 | 0 |
| Missing | 0 | 1 (<1) | 0 | 0 | 0 | 1 (<1) | 0 | 0 |
| Stage IV disease at diagnosis^e^ | 84 (70) | 267 (75) | 21 (62) | 72 (70) | 59 (66) | 282 (75) | 21 (60) | 72 (70) |
| Prior adjuvant chemotherapy | 21 (18) | 54 (15) | 9 (26) | 16 (16) | 16 (18) | 58 (15) | 10 (29) | 16 (16) |
| Kӧhne prognostic score |  |  |  |  |  |  |  |  |
| High | 7 (6) | 86 (24) | 1 (3) | 8 (8) | 4 (4) | 88 (23) | 1 (3) | 8 (8) |
| Medium | 76 (63) | 208 (59) | 18 (53) | 59 (57) | 60 (67) | 220 (59) | 19 (54) | 59 (57) |
| Low | 37 (31) | 56 (16) | 15 (44) | 36 (35) | 25 (28) | 62 (17) | 15 (43) | 36 (35) |
| Unclassifiable | 0 | 5 (1) | 0 | 0 | 0 | 5 (1) | 0 | 0 |
| Median neutrophil/lymphocyte ratio, (IQR) | 2.95 (2.17–4.18) | 3.67  (2.63–5.13) | NA | NA | 2.93  (2.35–4.06) | 3.63  (2.62–5.07) | NA | NA |
| Data are n (%), unless otherwise indicated.  ^a^Long-term survival defined as the 25% of classifiable patients with the longest OS (this included some patients that survived less than 45 months); all other patients were defined as short-term survivors.  ^b^Long-term survival defined as OS ≥45 months; short-term survival defined as OS <45 months.  ^c^PRIME: panitumumab arm = panitumumab +FOLFOX4; PEAK: panitumumab arm = panitumumab + modified FOLFOX6.  ^d^PRIME: comparator arm = FOLFOX4 alone; PEAK: comparator arm = bevacizumab + modified FOLFOX6.  ^e^Presence of stage IV disease at baseline was derived by taking date of metastases – date of primary diagnosis (allowing a 2-month window).  ECOG, Eastern Cooperative Oncology Group; IQR, interquartile range; NA, not available; OS, overall survival. | | | | | | | | |

| **Supplementary Table 2. Response to first-line therapy in long- and short-term survivors in PRIME and PEAK by tumour location (defined using the 25% survival cut-off)** | | | | | | | | | | | | |
| --- | --- | --- | --- | --- | --- | --- | --- | --- | --- | --- | --- | --- |
| **Characteristic** | **PRIME** | | | | | | **PEAK** | | | | | |
|  | **Long-term survivor** | | | **Short-term survivor** | | | **Long-term survivor** | | | **Short-term survivor** | | |
|  | Overall^b^ (n=120) | Left (n=92) | Right  (n=12) | Overall^b^  (n=355) | Left (n=218) | Right  (n=73) | Overall^b^  (n=34) | Left  (n=29) | Right  (n=3) | Overall^b^  (n=103) | Left  (n=56) | Right  (n=29) |
| Exposure duration, n (%) |  |  |  |  |  |  |  |  |  |  |  |  |
| <3 months | 5 (4) | 3 (3) | 0 | 72 (20) | 34 (16) | 21 (29) | 4 (12) | 3 (10) | 0 | 15 (15) | 7 (13) | 7 (24) |
| ≥3 to <6 months | 21 (18) | 16 (17) | 2 (17) | 110 (31) | 72 (33) | 26 (36) | 6 (18) | 6 (21) | 0 | 28 (27) | 15 (27) | 7 (24) |
| ≥6 to <9 months | 27 (23) | 20 (22) | 5 (42) | 83 (23) | 55 (25) | 10 (14) | 7 (21) | 5 (17) | 2 (67) | 23 (22) | 10 (18) | 7 (24) |
| ≥9 months | 67 (56) | 53 (58) | 5 (42) | 90 (25) | 57 (26) | 16 (22) | 17 (50) | 15 (52) | 1 (33) | 37 (36) | 24 (43) | 8 (27) |
| Resection, n (%) |  |  |  |  |  |  |  |  |  |  |  |  |
| Any | 36 (30) | 27 (29) | 5 (42) | 21 (6) | 14 (6) | 5 (7) | 9 (26) | 9 (31) | 0 | 3 (3) | 1 (2) | 1 (3) |
| Complete | 29 (24) | 22 (24) | 3 (25) | 9 (3) | 8 (4) | 0 | 6 (18) | 6 (21) | 0 | 3 (3) | 1 (2) | 1 (3) |
| Best overall response, n (%) |  |  |  |  |  |  |  |  |  |  |  |  |
| Complete response | 0 | 0 | 0 | 2 (1) | 2 (1) | 0 | 5 (15) | 3 (10) | 1 (33) | 0 | 0 | 0 |
| Partial response | 89 (74) | 69 (75) | 6 (50) | 160 (45) | 116 (53) | 23 (32) | 24 (71) | 22 (76) | 1 (33) | 59 (57) | 27 (48) | 17 (59) |
| Stable disease | 25 (21) | 19 (21) | 5 (42) | 136 (38) | 72 (33) | 36 (49) | 5 (15) | 4 (14) | 1 (33) | 32 (31) | 23 (41) | 7 (24) |
| Progressive disease | 4 (3) | 4 (4) | 0 | 44 (12) | 22 (10) | 11 (15) | 0 | 0 | 0 | 5 (5) | 4 (7) | 1 (3) |
| Not known/unavailable | 2 (2) | 0 | 1 (8) | 13 (4) | 6 (3) | 3 (4) | 0 | 0 | 0 | 7 (7) | 2 (4) | 4 (14) |
| ETS, n (%) | 77 (64) | 59 (64) | 6 (50) | 123 (35) | 92 (42) | 19 (26) | 26 (76) | 23 (79) | 2 (67) | 42 (41) | 19 (34) | 11 (38) |
| Median DpR (IQR), % | 75 (60–89) | 75 (61–84) | 75 (46–100) | 43 (18–59) | 45 (25–64) | 31 (12–52) | 86 (61–100) | 87 (61–100) | 75 (8–100) | 45 (26–60) | 45 (25–60) | 43 (20–60) |
| Received post-progression EGFRi therapy, n (%) | 44 (37) | 35 (38) | 3 (25) | 62 (17) | 34 (16) | 13 (18) | 16 (47) | 13 (45) | 2 (67) | 40 (39) | 23 (41) | 10 (35) |
| ^a^Long-term survival defined as the 25% of classifiable patients with the longest OS (this included some patients that survived less than 45 months); all other patients were defined as short-term survivors.  ^b^‘Overall’ columns include data for patients in whom primary tumour location could not be determined.  DpR, depth of response; EGFRi, epidermal growth factor receptor inhibitor; ETS, early-tumour shrinkage; IQR, interquartile range; OS, overall survival. | | | | | | | | | | | | |

| **Supplementary Table 3. Univariate analysis of long-term overall survival in PRIME and PEAK (45-month cut-off for long-term survival)** | |
| --- | --- |
|  | **Odds ratio (95% CI)^a^** |
| **PRIME** | |
| Köhne score |  |
| Low risk vs. high risk | 8.87 (2.94–26.76) |
| Medium risk vs. high risk | 6 (2.12–17.01) |
| Treatment: FOLFOX4 vs. panitumumab + FOLFOX4 | 0.51 (0.31–0.81) |
| Sites of metastases |  |
| Liver + other vs. liver only | 0.43 (0.24–0.75) |
| Other only vs. liver only | 0.25 (0.11–0.61) |
| BRAF status |  |
| Mutant vs. wild-type | 0.07 (0.01–0.53) |
| Missing vs. wild-type | 1.11 (0.30–4.12) |
| Region: Rest of the world vs. Western Europe, Canada or Australia | 0.66 (0.41–1.07) |
| Primary tumour location |  |
| Right vs. left | 0.46 (0.23–0.94) |
| Unknown vs. left | 0.51 (0.25–1.04) |
| Prior adjuvant chemotherapy: No vs. yes | 0.84 (0.45–1.54) |
| Age (≥65 years): No vs. yes | 1.27 (0.78–2.06) |
| Sex: Female vs. male | 0.86 (0.53–1.40) |
| Stage |  |
| I–III vs. IV | 1.56 (0.93–2.59) |
| Missing vs. IV | 1.37 (0.28–6.74) |
| ECOG performance status |  |
| 1 vs. 0 | 0.38 (0.23–0.66) |
| 2 vs. 0 | 0.05 (0.00–0.77)^b^ |
| **PEAK** | |
| Köhne score |  |
| Low risk vs. high risk | 3.33 (0.38–29.02) |
| Medium risk vs. high risk | 2.58 (0.30–21.94) |
| Treatment: mFOLFOX6 + bevacizumab vs. mFOLFOX6 + panitumumab | 0.52 (0.24–1.13) |
| Sites of metastases |  |
| Liver + other vs. liver only | 0.65 (0.25–1.66) |
| Other only vs. liver only | 0.73 (0.26–2.06) |
| BRAF status: Mutant vs. wild-type | 0.10 (0.01–1.79)^b^ |
| Primary tumour location |  |
| Right vs. left | 0.19 (0.05–0.69) |
| Unknown vs. left | 0.21 (0.05–0.96) |
| Prior adjuvant chemotherapy: No vs. yes | 0.46 (0.19–1.14) |
| Age (≥65 year): No vs. yes | 1.50 (0.67–3.39) |
| Sex: Female vs. male | 2.41 (1.09–5.31) |
| Stage |  |
| I–III vs. IV | 1.52 (0.66–3.52) |
| Missing vs. IV | 1.72 (0.29–10.02) |
| ECOG performance status: 1 vs. 0 | 0.38 (0.16, 0.92) |
| **PRIME and PEAK** | |
| Köhne score |  |
| Low risk vs. high risk | 7.84 (2.97–20.7) |
| Medium risk vs. high risk | 5.44 (2.14–13.82) |
| Treatment: Comparator vs. panitumumab | 0.51 (0.34–0.76) |
| Sites of metastases |  |
| Liver + other vs. liver only | 0.47 (0.29–0.75) |
| Other only vs. liver only | 0.40 (0.21–0.76) |
| BRAF status |  |
| Mutant vs. wild-type | 0.05 (0.01–0.39) |
| Missing vs. wild-type | 1.01 (0.27–3.74) |
| Primary tumour location |  |
| Right vs. left | 0.37 (0.20–0.69) |
| Unknown vs. left | 0.41 (0.22–0.79) |
| Prior adjuvant chemotherapy: No vs. yes | 0.69 (0.42–1.14) |
| Age (≥65 years): No vs. yes | 1.32 (0.87–2.01) |
| Sex: Female vs. male | 1.13 (0.75–1.70) |
| Stage |  |
| I–III vs. IV | 1.57 (1.01–2.41) |
| Missing vs. IV | 1.61 (0.5–5.18) |
| ECOG performance status |  |
| 1 vs. 0 | 0.38 (0.24–0.60) |
| 2 vs. 0 | 0.04 (0.00–0.72)^b^ |
| ^a^An odds ratio >1 indicates that long-term survival is more likely for the first parameter listed, while an odds ratio <1 indicates that long-term survival is more likely for the second parameter listed.  ^b^The logit estimator, which adds half-an-event for calculation of OR when there are zero counts, was used as there were no subjects with long-term survival and either an ECOG performance status of 2 or a *BRAF* mutant status.  CI, confidence interval; ECOG, Eastern Cooperative Oncology Group; mFOLFOX6, modified FOLFOX6. | |
